# Supplementary material for: Sequence-Dependent Correlated Segments in the Intrinsically Disordered Region of ChiZ
Source: Biomolecules. 2020 Jun 23;10(6):946. doi: 10.3390/biom10060946 (PMC7355643; doi:10.3390/biom10060946)
Supplement: Supplementary file 1 [file biomolecules-10-00946-s001.pdf]

Sequence-dependent correlated segments in the intrinsically disordered region of ChiZ

Alan Hicks [1,2,4,†], Cristian A. Escobar[1,3,4,†], Timothy A. Cross [1,3,4,*] and Huan-Xiang Zhou [5,*]

[1] Institute of Molecular Biophysics, Florida State University, Tallahassee, Florida 32306, United States
[2] Department of Physics, Florida State University, Tallahassee, Florida 32306, United States
[3] Department of Chemistry and Biochemistry, Florida State University, Tallahassee, Florida 32306, United States
[4] National High Magnetic Field Laboratory, Florida State University, Tallahassee, Florida 32310, United States
[5] Department of Chemistry and Department of Physics, University of Illinois at Chicago, Chicago, Illinois 60607, United States
* Correspondence: Timothy A. Cross, cross@magnet.fsu.edu; Huan-Xiang Zhou, hzhou43@uic.edu
† Denotes equal contribution

Received: date; Accepted: date; Published: date

Supporting Information: 11 figures, 1 table

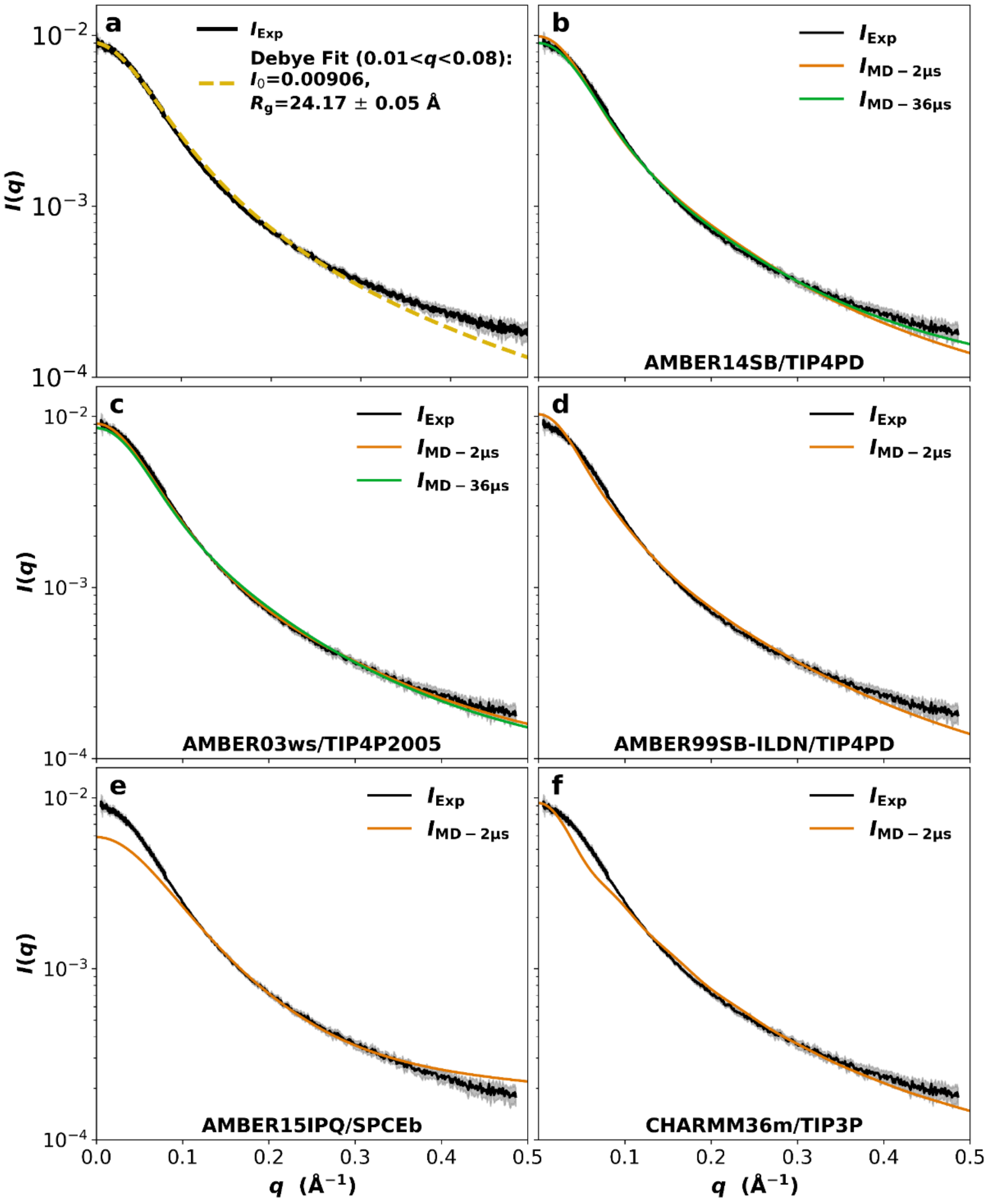

Figure S1. Measured and predicted SAXS profiles. (a) Experimental SAXS profile and Debye fit. (b-f) SAXS profiles calculated from conformations sampled in MD simulations using five different force fields, are compared with the experimental counterpart.

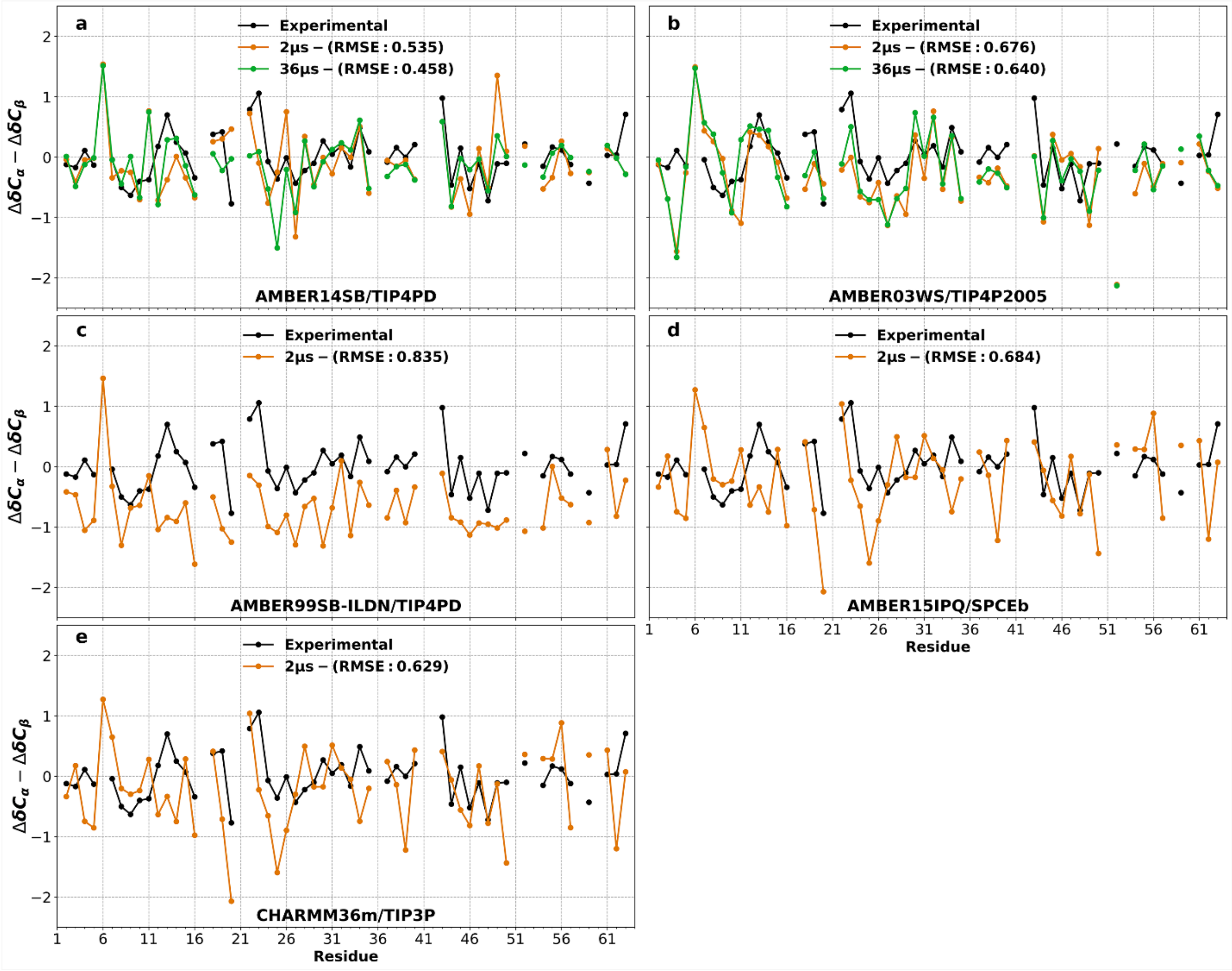

Figure S2. Measured and predicted chemical shifts.

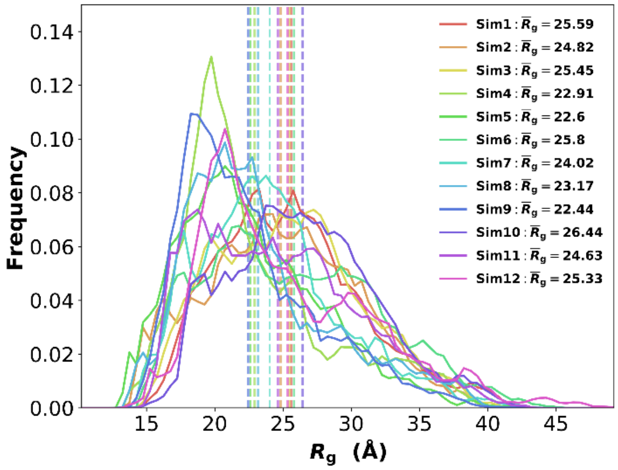

Figure S3. Radii of gyration (R_g) from 12 replicate simulations.

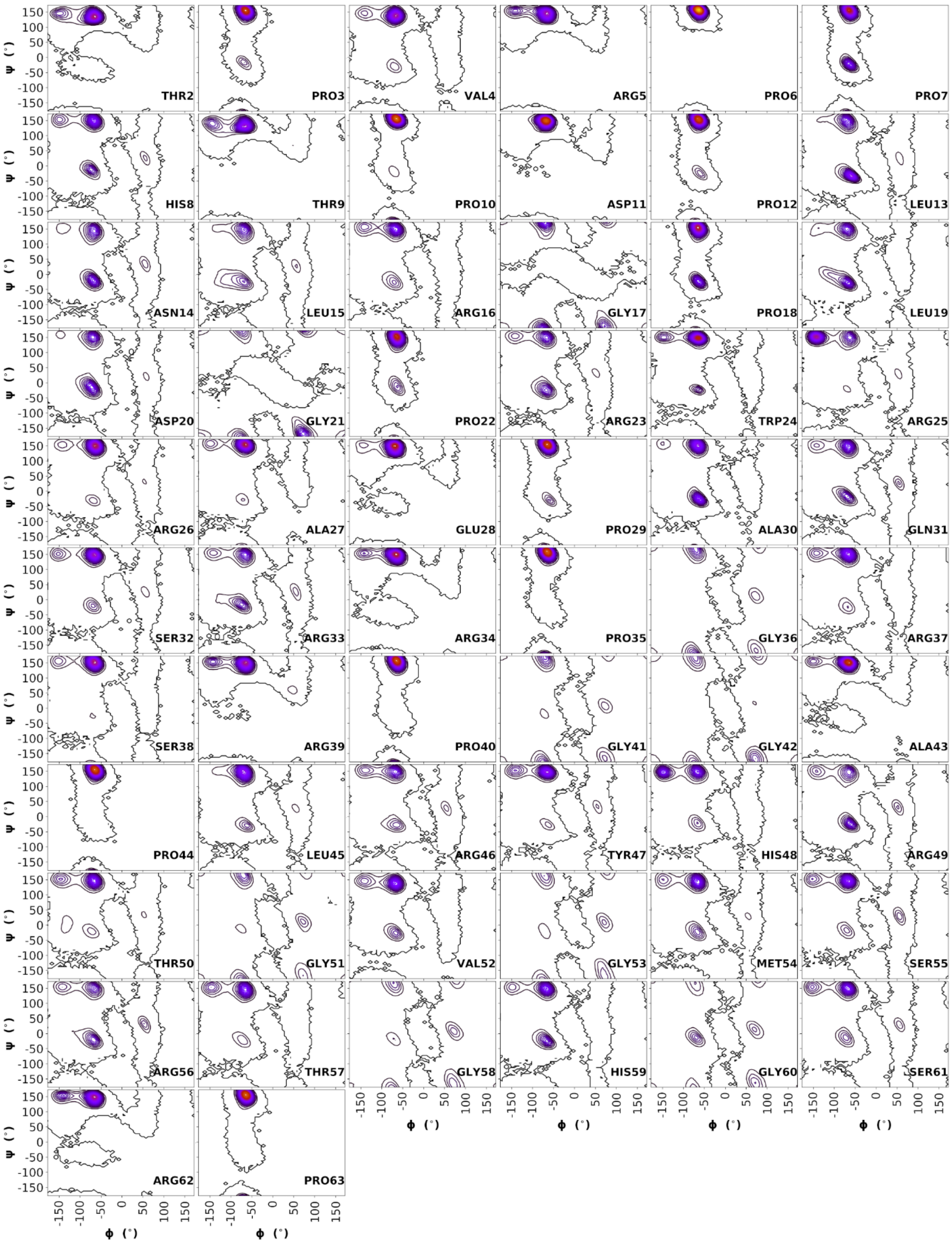

Figure S4. Ramachandran maps for the 62 non-terminal residues in ChiZ1-64. Frequencies in descending order are presented by contours in colors from yellow to red to purple.

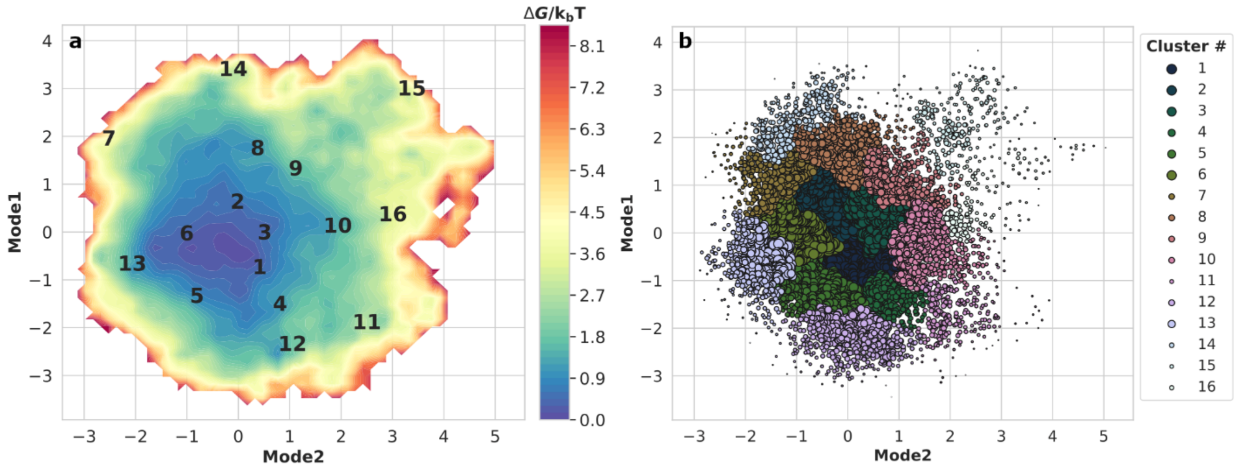

Figure S5. Dihedral princial component analysis (dPCA) and clustering. (a) The density of MD conformations in the subspace of the projections along the first two dPCA modes, converted to a free energy surface. (b) Clustering of conformations according to the projections along the first two modes. The 16 clusters are separted by color; circle sizes are proportional to the density. The numbers in (a) are cluster numbers, and take positions from the representative conformations (shown in Figure 4) selected from the clusters.

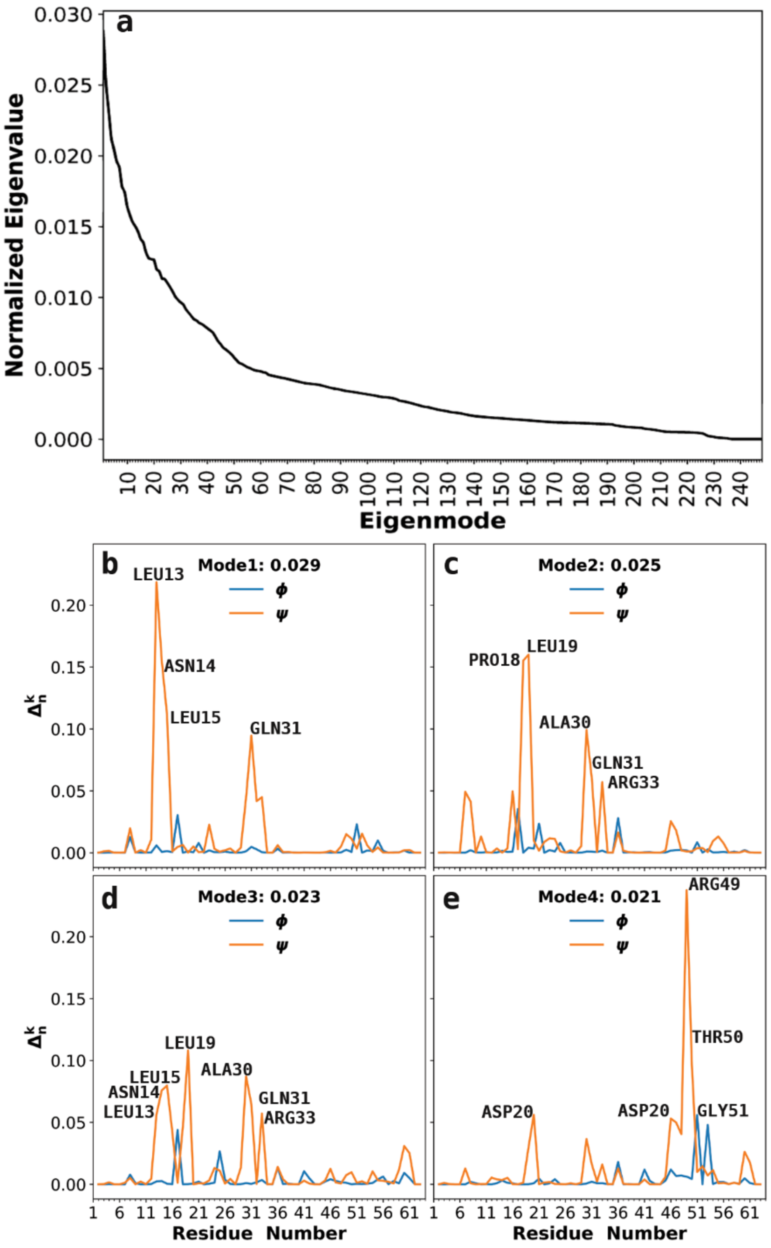

Figure S6. Eigenvalues and eigenmodes from dPCA. (a) Normalized eigenvalues. (b-e) Amplitudes of torsion angles in the first four modes. Residues with greater than 5% contributions to a mode are labeled. In the legend, the normalized eigenvalue is shown.

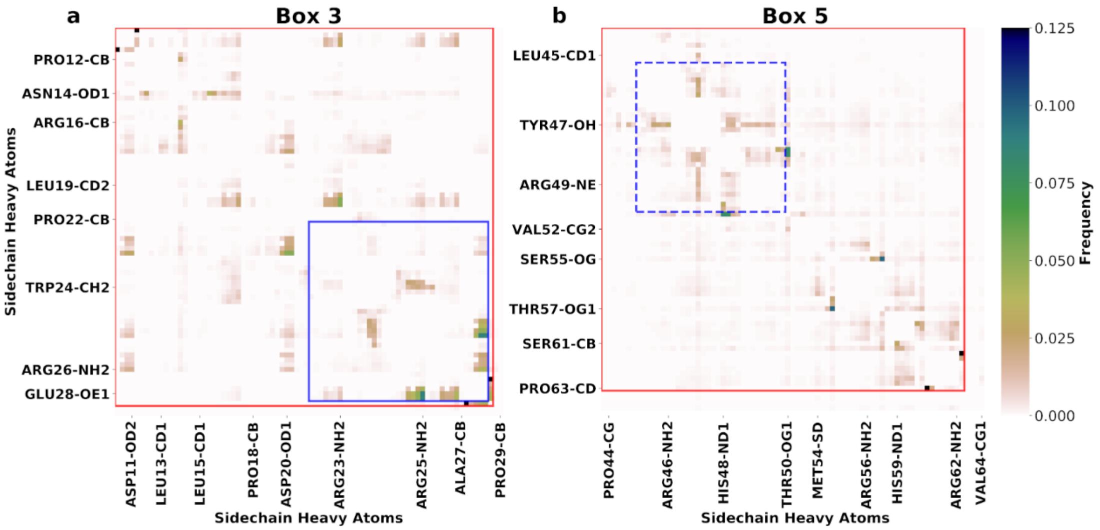

Figure S7. Enlarged view of Box 3 and Box 5 from the contact maps shown in Figure 5.

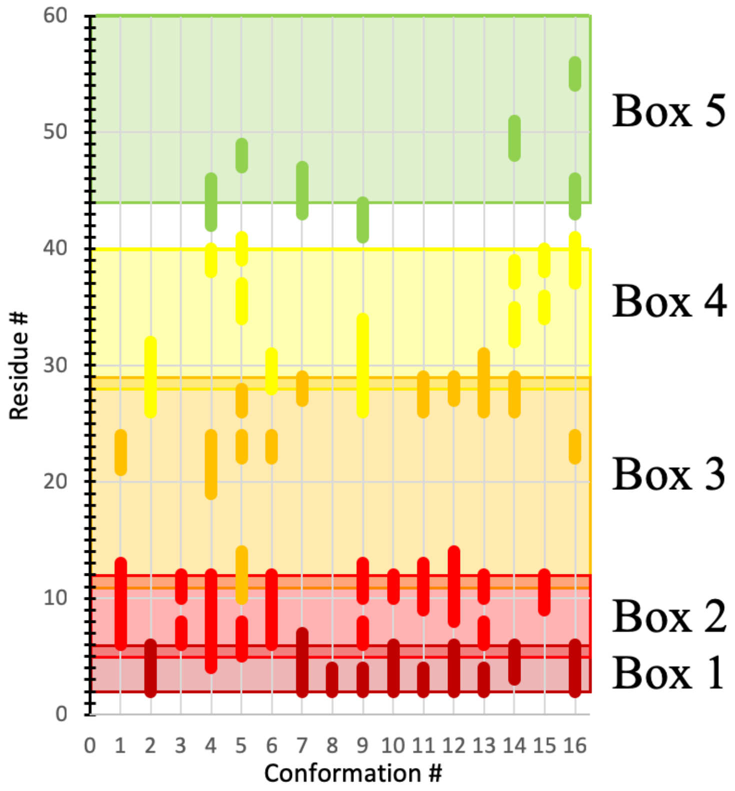

Figure S8. PPII stretches in the 16 conformations in Figure 4. Each PPII stretch is displayed by a vertical bar extending from the beginning to the ending residues. Five sequence segments, corresponding to Box 1 to Box 5 in Figure 5, are indicated by shaded boxes. The color of each PPII bar is selected according to the box that contains all (or the most) of the residues in the PPII stretch.

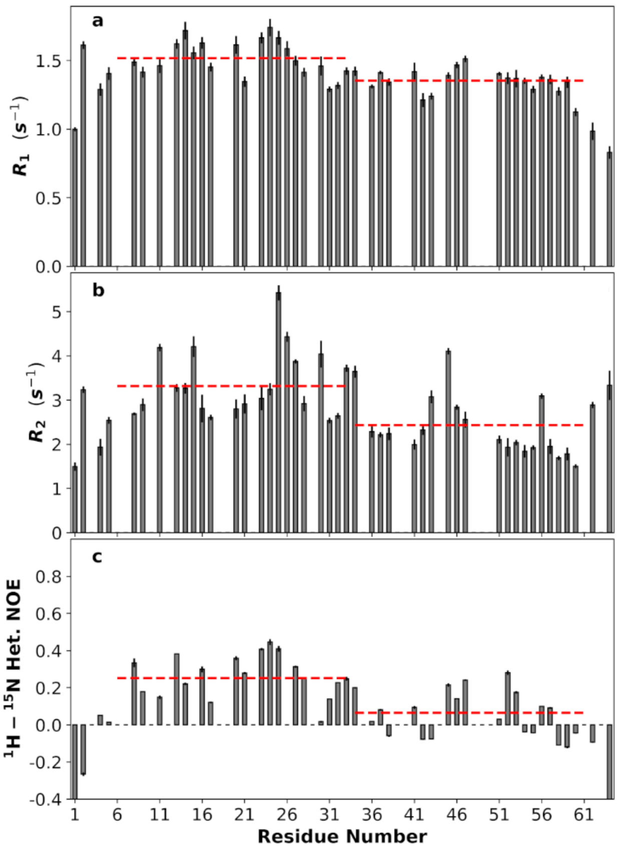

Figure S9. NMR relaxation parameters (R_1, R_2, and NOE) at pH 4.0. Red dashes indicate averages over residues 5-32 and 33-60.

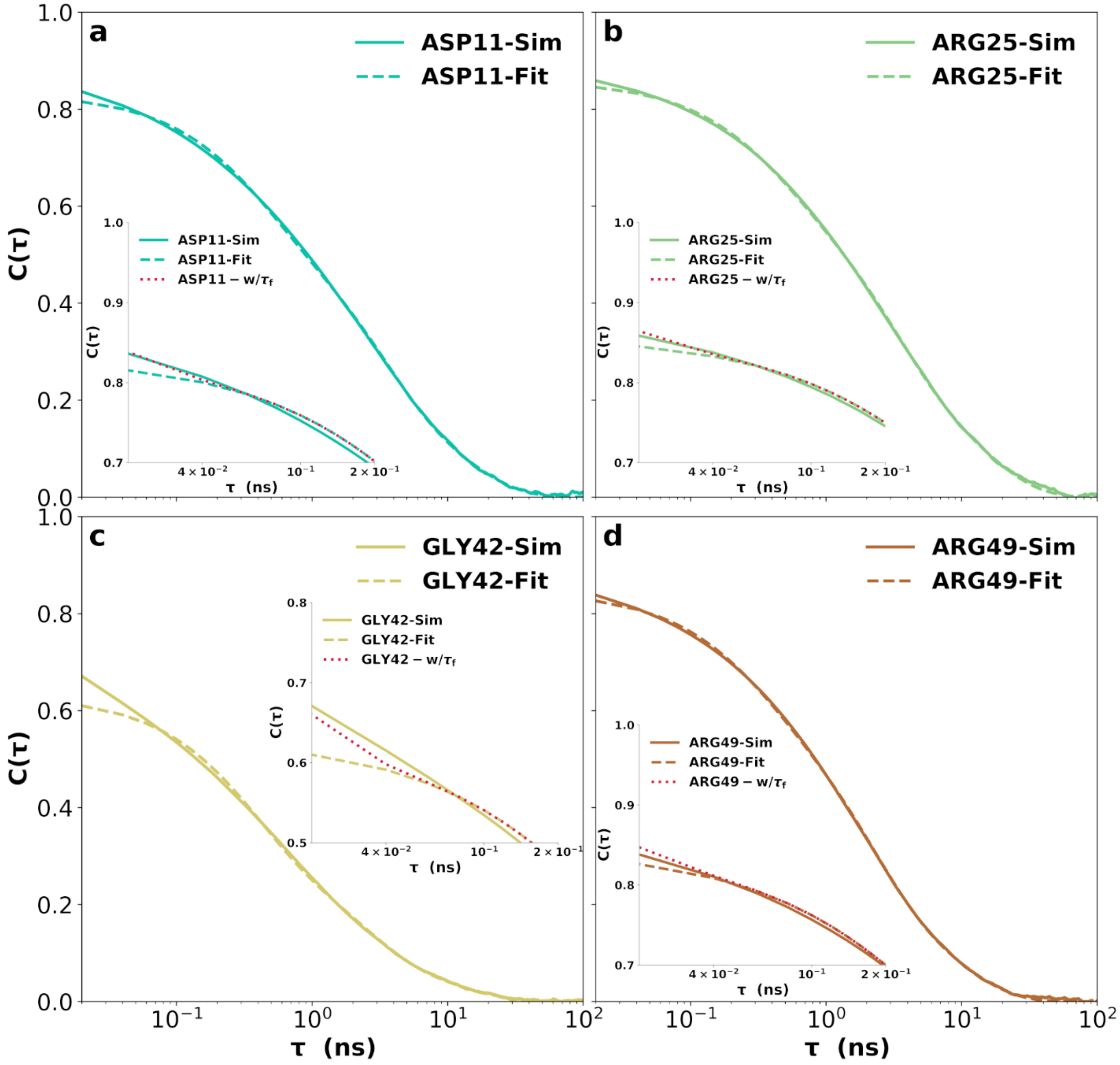

Figure S10. Representative correlation functions and tri-exponential fits. (a) Asp11; (b) Arg25; (c) Gly42; (d) Arg49. Insets show in red the addition of the ultrafast decay (with time constant τ_f = 10 ps) to the correlation functions.

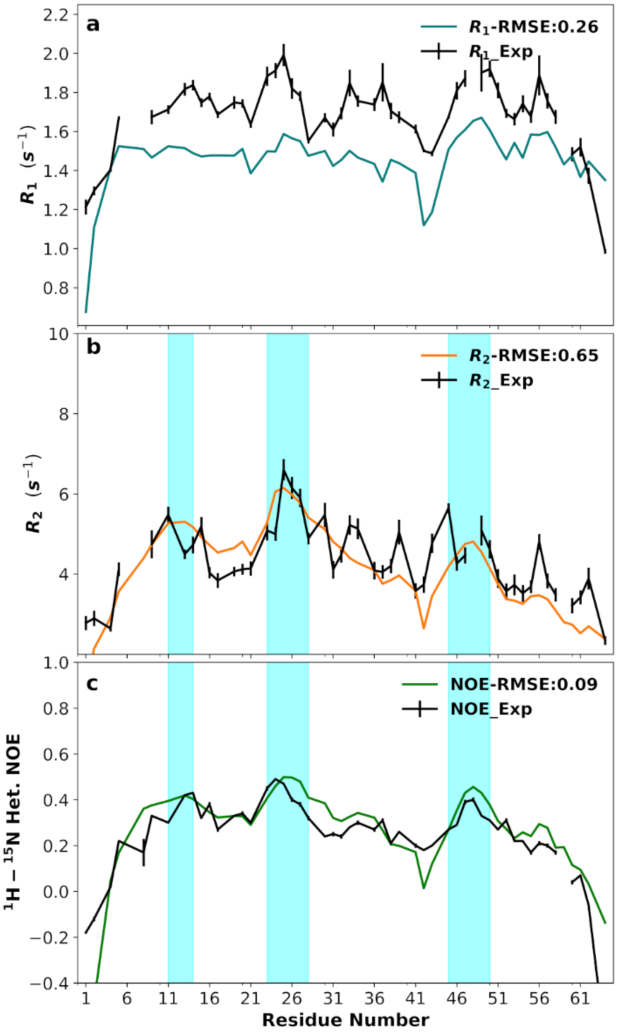

Figure S11. Backbone ^{15}N relaxation parameters from MD simulations, after modifications to include ultrafast decay and tempered slow dynamics.

Table S1. p-values and t-statistics comparing the means of the two halves of ChiZ1-64 for NMR relaxation at pH 7.0 and pH 4.0 and for amide proton exchange at pH 7.0, according to the independent-samples t-test assuming unequal variances.

	**pH 7.0**		**pH 4.0**	
	p-value	t-statistic	p-value	t-statistic
R_1	0.57	0.57	5.85E-05	4.61
R_2	0.013	2.62	5.18E-05	3.79
**NOE**	0.0014	3.43	6.27E-05	4.53
**Protection Factor**	0.015	2.65		
